# Novas disputas e intervenções no corpo feminino: fronteiras entre ginecologia e cirurgia plástica

**DOI:** 10.1590/S0104-59702024000100006

**Published:** 2024-04-05

**Authors:** Fabíola Rohden

**Affiliations:** Professora, Programa de Pós-graduação em Antropologia Social/Universidade Federal do Rio Grande do Sul. Porto Alegre – RS – Brasil fabiola.rohden@gmail.com

**Keywords:** Genital surgery, Female genital esthetics, Gynecology, Aesthetic surgery, Gender, Cirurgia íntima, Estética genital feminina, Ginecologia, Cirurgia estética, Gênero

## Abstract

Este artigo analisa tensões e disputas entre o campo da ginecologia e da cirurgia plástica estética, especialidades autorizadas a realizar a cirurgia estética genital feminina no Brasil. Utiliza material documental, incluindo artigos científicos desde a década de 1990, e *sites* institucionais. Enquanto ginecologistas têm se mantido mais cautelosos com a prática, defendendo sua realização apenas quando há indicações funcionais, cirurgiões/ãs plásticos/as têm sido mais influentes na disseminação do procedimento, privilegiando a dimensão estética. Argumenta-se que, para além de disputas entre campos profissionais, esse fenômeno precisa ser entendido à luz da crescente ênfase no aprimoramento de si, via recursos biomédicos, e dos imperativos de gênero.

## Abstract (en)

This article analyzes the tensions and disputes between the fields of gynecology and esthetic plastic surgery, the specialties which are authorized to perform aesthetic female genital surgeries in Brazil. Documentary materials are used, including scientific articles from the 1990s onward and institutional websites. While gynecologists have remained more cautious, recommending the surgery only for functional reasons, plastic surgeons have been more influential in publicizing these procedures and emphasizing the aesthetic dimension. Beyond professional disputes, we debate whether this phenomenon needs to be understood in light of the growing emphasis on self-improvement via biomedical resources and gender imperatives.

O vasto campo dos procedimentos médicos dedicados ao aprimoramento estético, especialmente das mulheres, continua em crescimento e adentrando áreas que parecem, à primeira vista, inusitadas. Esse parece ser o caso das chamadas cirurgias estéticas genitais em mulheres cisgênero. A produção desse terreno das cirurgias “íntimas”, e especialmente da intervenção mais comum, a chamada ninfoplastia, ou cirurgia de redução dos pequenos lábios, não se constitui sem uma série de controvérsias surgidas ao longo do tempo. Pretendo, neste artigo, trazer à tona algumas delas que, de forma particular, incidem sobre as tensões entre o campo da ginecologia e da cirurgia plástica, especialidades autorizadas pelo Conselho Federal de Medicina a realizar as cirurgias genitais, e sobre as fronteiras entre o que seriam intervenções de ordem estética e funcional. Certamente, há muitas diferenças na história e na prática atual dessas duas especialidades, uma especialmente dedicada aos problemas relativos ao corpo feminino e aos órgãos sexuais e reprodutivos (Moscucci, 1996; Rohden, 2001), e a outra comprometida com a reparação ou melhoria estética do corpo e sua aparência (Gilman, 2001; Edmonds, 2010; Jarrín, 2017). São dois campos profissionais bastante distintos, o que justificaria outra análise, mas que vão ser considerados aqui exclusivamente por meio de sua intercessão na prática das cirurgias íntimas. Para tanto, serão analisados artigos científicos e outros documentos que permitem apreender os contrastes entre essas duas especialidades e a constituição desse novo foco de intervenções no corpo feminino.

De acordo com os dados da Sociedade Internacional de Cirurgia Plástica Estética (Isaps, 2019), referentes a procedimentos realizados em 2019, o Brasil é o país campeão no número de procedimentos estéticos cirúrgicos, chegando a 1.493.673 (13,1%) e ultrapassando até mesmo os EUA, onde se realizaram 1.351.917 (11,09%) intervenções, do total de 11.363.569 efetuadas no mundo. Também é aqui onde mais se realiza a ninfoplastia. Foram 30.356 (18,43%), em comparação com 12.006 (7,29%) nos EUA, considerando o total de 164.667 cirurgias realizadas no mundo. Na maioria dos casos, essas intervenções foram realizadas por um/a cirurgião/ã plástico/a (73,3%).

Quanto à procura por tal procedimento, não há dados oficiais. Contudo, por meio das observações clínicas relatadas em artigos de cirurgiões/ãs plásticos/as, o número total de procedimentos realizados por profissional variava entre três e 469, e a faixa etária dos 12 aos 70 anos, com a média girando em torno dos 30 anos (Rohden, Cavalheiro, 2021). Considerando as matérias na imprensa e a pesquisa sobre a temática em redes sociais, junto a grupos em Facebook, Whatsapp e *sites* que oferecem serviços de informação sobre cirurgias estéticas, é possível dizer que mulheres jovens, mas de várias idades e de diversificado pertencimento social e étnico, têm se interessado pelo procedimento. A maior parte das cirurgias ocorre no sistema privado de saúde e tem custo elevado. No sistema público e também nos planos de saúde, é necessário apresentar uma justificativa atrelada à funcionalidade do procedimento.

Este trabalho se ancora, por um lado, nos estudos sociais da ciência e gênero que vêm produzindo uma perspectiva crítica à produção do conhecimento científico e, em especial, da medicina e práticas médicas. Autoras como Haraway (1995), Fausto-Sterling (1992), Bleier (1997), Birke (1986), Oudshoorn (1994), Rocha, Rocha e Keller (2022) há muito têm atentado para o modo como a produção do conhecimento biológico e biomédico, especialmente acerca das diferenças marcadas nos corpos, sustenta-se e ajuda a sustentar a própria hierarquia entre os gêneros.

Por outro lado, são centrais as referências sobre a cirurgia plástica estética e, especialmente, os estudos que têm mapeado esse campo no Brasil, considerando seus processos de institucionalização, mas, sobretudo, discutindo o impacto dos marcadores de gênero, classe e raça/etnia e as fronteiras entre saúde e aprimoramento estético. A literatura tem apontado para como, no contexto brasileiro, uma noção de saúde associada à ideia geral de qualidade de vida, autoestima e bem-estar tem justificado as intervenções cirúrgicas estéticas como associadas à promoção da saúde. Além disso, a valorização de certos padrões de beleza e sua distinção associada à classe e à raça/etnia contribuem para a produção de hierarquias e chances desiguais de mobilidade social. Trata-se de constatações que caracterizam processos de normalização do desejável, especialmente nos corpos femininos (Edmonds, 2007, 2009, 2010, 2013; Edmonds e Sanabria, 2014, 2016; Jarrín, 2017).

Nesse contexto, as cirurgias íntimas podem ser apresentadas como um caso particular no qual as exigências em torno de um corpo feminino “perfeito”, e alvo de grandes investimentos, tornam-se imperativas e conduzem a transformações corporais e subjetivas importantes (Silva, 2019; Rohden, 2021; Rohden, Cavalheiro, 2021). Isso tem ocorrido, sobretudo, a partir do crescente impacto da internet e das redes sociais, que têm configurado novas formas de apresentação de si, tipos de sociabilidade e mesmo publicização de imagens que ilustram os contornos corporais ideais a ser buscados (Rohden, Silva, 2020).

## Definições iniciais de uma nova prática

Como uma forma de introduzir o cenário dessas disputas, recorro a uma das referências mais antigas publicadas sobre o tema no Brasil. Trata-se, curiosamente, de um artigo escrito por dois cirurgiões plásticos, mas publicado no *Jornal Brasileiro de Ginecologia*, periódico vinculado à Associação de Ginecologia e Obstetrícia do Estado do Rio de Janeiro. Esse trabalho pioneiro sobre o tema (Franco, Franco, 1993) tornou-se referência no campo. A ausência de outros artigos nesse período, tanto em revistas de ginecologia como de cirurgia plástica, é um indício de como o tema das cirurgias íntimas só passa a ganhar a atenção, inclusive dos ginecologistas, mais recentemente.

O artigo “Hipertrofia de ninfas” foi escrito por Talita Franco e Diogo Franco^1^ (1993) e pode ser entendido como uma tentativa de diálogo entre as áreas da ginecologia e da cirurgia plástica. Serve também aqui para introduzir a temática das cirurgias íntimas. O texto tem início com a seguinte afirmação:

As alterações de forma e/ou dimensões das estruturas presentes na genitália externa feminina são comuns e, dentro de certos limites, não caracterizam anormalidades. Entre elas, a hipertrofia dos pequenos lábios vulvares tem sido relatada com certa frequência, sendo, na maioria das vezes, discreta, mas podendo, ocasionalmente, trazer algum tipo de desconforto para a paciente (Franco, Franco, 1993, p.163).

A autora e o autor observam que a procura por soluções cirúrgicas era pequena, bem como era “escasso” o número de publicações a respeito. Acrescentam que “é curioso observar que o tratamento dessa dismorfia ‘eminentemente ginecológica’ vem sendo feito mais frequentemente pelo cirurgião plástico” (Franco, Franco, 1993, p.163; destaque meu). Supõem, como razões para isso, que

o ginecologista, mais afeito à anatomia da região, não valorize pequenas variações que ainda podem ser consideradas dentro da normalidade e as pacientes se sintam constrangidas em insistir. Para o cirurgião plástico, que lida com o detalhe e sabe a importância que pode representar, a cirurgia é vista com naturalidade (Franco, Franco, 1993, p.163).

No final do artigo, Franco e Franco (1993, p.166) voltam a esse contraste entre as duas especialidades, afirmando que a pequena incidência da cirurgia de redução das ninfas entre ginecologistas é intrigante e talvez tivesse a ver mais com “o silêncio das pacientes”. Acrescentam que a função do ginecologista “é cuidar da saúde dos genitais, e não de sua estética, e nem lhes caberia perguntar se a paciente está satisfeita com o tamanho de suas ninfas ou se deseja reduzi-las” (p.166). Mas também questionam se para alguns ginecologistas a cirurgia puramente estética seria justificável. Essa dúvida faria as pacientes procurarem diretamente um cirurgião plástico “que nada achará de estranho em seu desejo, pela própria natureza do trabalho que realiza” (p.166).

Os autores definem os pequenos lábios, *labia minora*, ou ninfas, como “duas pregas orientadas no mesmo sentido dos grandes lábios e situadas internamente a eles, medindo geralmente 30 a 35mm de comprimento por 10 a 15mm de largura” (Franco, Franco, 1993, p.163). Mencionam que “[d]iz-se que podem alcançar medidas exageradas em certas raças exóticas, constituindo o ‘véu do pudor’ ou ‘cortina dos hotentotes’”. Além disso, atestam que a membrana de revestimento das ninfas apresenta como características: “Coloração rósea, aspecto liso e úmido, ausência de pelos, de glândulas sudoríparas e de camada gordurosa subjacente, como as mucosas” (p.163-164). Nesse ponto, já percebemos como representações associadas não somente às diferenças de gênero, mas também de raça/etnia e valorização da juventude passam a ser inscritas na aparência da genitália (Nurka, 2019).

O artigo se destaca também por trazer referências mais antigas acerca de “algum tipo de hipertrofia vulvar” e cita trabalhos da passagem dos anos 1970 para 1980 que tratam da “epidemiologia” das mutilações genitais na África, explicando que esses “hábitos regionais” vinham chamando a atenção para esse tipo de prática que, por vezes, implicaria “mutilações extensas”. Por fim, referem-se ao trabalho de Hodgkinson e Hait (1984), que já apresentava três casos de cirurgia com indicação “predominantemente estética”.

É significativo o fato de que os autores estejam cotejando no geral mais de 20 anos de experiência no campo e só tenham realizado seis ninfoplastias, três delas entre 1991 e 1992, sendo que “[u]ma delas era homossexual” (Franco, Franco, 1993, p.166), observação que não se conecta com nenhum outro comentário. Ou seja, trata-se de um contingente muito pequeno de pacientes durante um longo período. Mas os autores já observam algumas mudanças, como o fato de que as últimas pacientes procuraram a cirurgia por motivos “puramente estéticos”, e as duas últimas eram de “camadas sociais mais baixas”, o que os leva a dizer que “[o] ideal de beleza e equilíbrio bem como o desejo de aceitação social e sexual já não são luxo das classes altas, mas sim direito de todos” (p.166-167).

Na parte final do trabalho, fazem algumas ponderações acerca de que, apesar da prática sexual ser mais precoce e frequente, a falta de intimidade com a própria genitália ainda seria uma “ocorrência comum”. Acrescenta-se a isso o fato de que “[m]eninas não têm o hábito, tão comum entre os meninos, de comparar os genitais. Não há parâmetros para saber o que é normal, grande ou pequeno, e alterações discretas podem assustar as portadoras” (Franco, Franco, 1993, p.166). Há, portanto, por um lado, uma valorização do autoconhecimento e da intimidade e, por outro, referências de comparação e mesmo de “normalidade”.

Por meio da revisão apresentada, concluem que “a cirurgia das ninfas já é feita de longa data, porém sempre com uma ‘conotação funcional’, como se percebe na bibliografia” (Franco, Franco, 1993, p.166; destaque meu). Nas conclusões finais, atestam que essa cirurgia não era uma ocorrência comum, mas que “a evolução dos costumes, tornando a mulher mais afeita à própria anatomia e mais ativa na esfera sexual, tende a tornar esta cirurgia mais frequente” (p.167). Não sabemos bem o que significaria “mais afeita à própria anatomia”, mas o certo é que a previsão acabou por se realizar. E, para tanto, afirmações como a última frase do texto devem ter contribuído: “É um procedimento simples que pode ser realizado sob qualquer tipo de anestesia (geral, peridural ou local) e resolve, adequada e definitivamente, as queixas das pacientes” (p.167). Esse ponto traduz a dimensão técnica envolvida, que indicaria uma suposta simplicidade e precisão do procedimento, posteriormente muitas vezes enfatizada, sobretudo no campo da cirurgia plástica.

Dessa forma, o trabalho de Franco e Franco (1993) já introduz algumas das principais questões ou contrastes que seguirão se adensando ao longo do tempo: a diferença entre cirurgiões plásticos e ginecologistas, entre estética e reparação ou funcionalidade, e também entre práticas distantes, hábitos regionais, que beiram a mutilação e os procedimentos, mesmo que puramente estéticos, realizados no âmbito da cirurgia estética. Se recorrermos às principais referências citadas na bibliografia internacional, podemos perceber alguns argumentos semelhantes.

## Referências pioneiras em um campo incipiente

O trabalho frequentemente citado como referência inaugural no campo, e que pode ser tomado como ilustração das perspectivas e questões correntes em meados dos anos 1980, é o artigo de poucas páginas sobre labioplastia vaginal estética dos cirurgiões plásticos Hodgkinson e Hait (1984).

O texto começa afirmando que as “pressões sociológicas” aplicadas às mulheres são muito difusas, incluindo anúncios que promovem uma silhueta magra, atlética, musculosa e sem gordura, e os serviços oferecidos para tal, como academias de ginásticas, centros de bem-estar, nutricionistas, cirurgiões plásticos, chamados a “refinar” o torso feminino tal qual o “ideal” desejado pelo grupo consumidor. Além disso, acrescenta que a exposição dos genitais femininos em revistas populares “permite uma avaliação mais crítica da estética genital feminina por homens e mulheres” (Hodgkinson, Hait, 1984, p.414).^2^ Considerando essas primeiras frases, temos a impressão de que o artigo apresentaria uma perspectiva algo crítica em relação às cirurgias. Na sequência, contudo, afirma-se, sem maiores discussões, que a cirurgia estética dos genitais externos pode ser legitimamente requerida por mulheres que desejam aprimorar sua estética e o prazer sexual.

O interessante é que o tópico seguinte a essa introdução chama-se “Circuncisão feminina: perspectivas históricas”. Aqui se usa uma bibliografia de referência acerca da “epidemiologia” da mutilação genital feminina em países da África e se faz referência ao fato de que, no Egito, cerca de 75% das mulheres teriam passado pela “circuncisão”, e somente 15% teriam sido feitas por um médico. Mas essa realidade estaria mudando, já que mulheres mais “modernas e educadas” estariam menos frequentemente permitindo a prática em suas filhas.

Na sequência, são relatados três casos de cirurgias realizadas pelos autores, incluindo a descrição breve das técnicas utilizadas e das fotos de pré e pós-operatório (que se tornariam referência depois). Nos três casos, foram cirurgias por demandas estéticas das pacientes, “mulheres de meia-idade” (duas de 30 e uma de 36 anos), sendo que duas delas procuraram o cirurgião inicialmente para a realização de uma mamoplastia de aumento, e as duas intervenções foram realizadas conjuntamente. Uma informação destacada é o fato de que as três teriam procurado inicialmente ginecologistas que se recusaram a realizar as operações. Na discussão final esse tema é retomado. Apesar de afirmar que a “hipertrofia” dos pequenos lábios tem sido reconhecida como uma “variante normal”, a “excisão do tecido redundante” vem sendo recomendada (Hodgkinson, Hait, 1984, p.416). Como ilustrado pelos três casos apresentados, porém, os ginecologistas vinham relutando em realizar a operação que, para os cirurgiões plásticos, aparece como parte do trabalho com o qual estão acostumados e para o qual estariam preparados.

Já no final da década de 1990, temos o também muito citado artigo de Alter (1998), cirurgião plástico que se aproxima das posições gerais de Hodgkinson e Hait (1984). O autor afirma que, naquele momento, a cirurgia estética da genitália feminina ainda não despertava muito interesse entre os médicos, apesar de as mulheres se tornarem cada vez “mais conscientes” das diferenças em função de imagens de nus em revistas e filmes. Também menciona que os/as ginecologistas se recusavam a fazer a cirurgia em mulheres que tomavam consciência da sua “deformidade labial leve” e desejavam realizar o procedimento.

Os artigos de Franco e Franco (1993), Hodgkinson e Hait (1984) e Alter (1998) ilustram alguns aspectos em comum na avaliação desses especialistas, nas últimas décadas do século XX. São cirurgiões plásticos afirmando que ainda havia uma baixa procura pelas cirurgias íntimas, mas com tendência a aumentar, em função do fato de as mulheres estarem se tornando cada vez mais “conscientes” e exigentes em relação aos seus corpos. E que as expectativas de transformação não eram consideradas ou atendidas pelos seus ginecologistas, dedicados prioritariamente ao aspecto funcional da genitália, em contraste com os cirurgiões plásticos, mais afeitos a reconhecer como, por vezes, um pequeno detalhe corporal poderia afetar a autoestima das suas pacientes.

Além disso, é curioso que nesses trabalhos mais antigos sejam comuns a referência à mutilação genital feminina e a menção à literatura médica sobre o tema. Talvez porque ainda não houvesse uma literatura mais específica sobre as cirurgias genitais femininas a ser citada. Gostaria, contudo, de chamar a atenção para o fato de que a menção e, portanto, alguma forma de aproximação com a mutilação genital deixa de estar presente nas referências futuras. E, mais do que isso, parece haver um esforço sistemático em separar os procedimentos médicos das práticas tradicionais, sobretudo as realizadas em sociedades africanas. Nesse ponto, vale mencionar que nesses artigos as práticas tradicionais eram rotuladas de “culturais”, apontando para uma exotização desses costumes. Já as práticas cirúrgicas no contexto da medicina ocidental não ganham o mesmo adjetivo. Sem querer entrar aqui na extensa discussão sobre a mutilação genital feminina, tão bem apresentada em outros trabalhos (como no mapeamento feito por Nurka, 2019), e o importante debate sobre as escolhas relativas das mulheres em diferentes contextos, gostaria de enfatizar a mudança de tom acionada pelos/as médicos/as. Diferentemente do que aparecia nesses artigos pioneiros sobre o tema, cada vez mais, em posicionamentos mais recentes, evita-se a justaposição entre essas duas práticas e contextos.

## Disputas no cenário brasileiro contemporâneo

Voltando ao contexto brasileiro, mais próximo, passo agora a analisar de que forma o tema aparece sob os auspícios das duas grandes sociedades médicas envolvidas nessa prática: a Sociedade Brasileira de Cirurgia Plástica (SBCP) e a Federação Brasileira das Associações de Ginecologia e Obstetrícia (Febrasgo). Para tanto, recorro especialmente ao material referenciado nas páginas oficias das duas instituições.

Conforme a apresentação que consta em seu *site*, a SBCP é uma das maiores associações mundiais da especialidade e órgão oficial da Associação Médica Brasileira e do Conselho Federal de Medicina, responsável por conferir o título de Especialista em Cirurgia Plástica.^3^ É importante salientar que os cirurgiões plásticos são responsáveis pela realização da maioria das cirurgias estéticas íntimas (Isaps, 2019). Quando, entretanto, procuramos informações e posicionamentos oficiais sobre categorias como plástica vaginal, cirurgias íntimas, cirurgia dos pequenos lábios, ninfoplastia ou labioplastia, encontramos apenas nove referências, sendo duas repetições e todas no *link* de “notícias”, sem discussões mais aprofundadas sobre o tema.

Em geral, as matérias publicadas entre 2012 e 2017 (SBCP, 5 nov. 2012, 25 nov. 2013, 30 abr. 2014, 11 ago. 2014, 18 abr. 2016, 20 maio 2016, 24 maio 2016, 26 dez. 2016, 18 abr. 2017) citam o aumento na procura por esse tipo de intervenção, dentro de um rol mais abrangente de busca por melhoramentos estéticos em geral. O texto que define o que é a ninfoplastia é publicado três vezes (uma em 2013 e duas em 2014), com o seguinte conteúdo:

A Ninfoplastia, também conhecida como cirurgia íntima, é a cirurgia plástica de redução dos pequenos lábios vaginais. Durante a puberdade, as mulheres podem ter um desenvolvimento anormal dos pequenos lábios vaginais, adquirindo tamanho desproporcional que por vezes tornam-se maiores que os grandes lábios. Além do desconforto estético pelo tamanho excessivo, o problema pode causar danos de ordem funcional, dificultando a higiene, a ventilação do local e trazendo incômodo durante as relações sexuais. A ninfoplastia é a cirurgia responsável pela retirada do excesso de mucosa do local e por devolver o aspecto natural da genitália feminina (SBPC, 25 nov. 2013, s.p.).

E como exemplo das outras matérias reproduzidas e, portanto, referendadas pela SBCP, é possível citar a notícia publicada em 5 de novembro de 2012, afirmando que as intervenções estéticas têm se tornado comuns na vida dos brasileiros e são usadas cada vez mais para corrigir ou melhorar várias partes do corpo, inclusive as partes íntimas (SBCP, 5 nov. 2012). A matéria segue fazendo referência ao depoimento do então presidente da SBCP, Sebastião Nelson Edy Guerra, que afirmou que o número de pacientes em busca das cirurgias íntimas havia aumentado cerca de 50% nos últimos dois anos. O médico explica essa procura nos seguintes termos: “Cada vez mais, a mulher deseja satisfação pessoal e liberdade. Antigamente, essa questão ficava escondidinha e ‘elas só falavam com o ginecologista’. Mas hoje, com a conquista da maior liberdade sexual e independência, houve esse espaço para a mulher melhorar e buscar perfeição em alguns pontos” (SBCP, 5 nov. 2012, s.p.; destaque meu).

O cirurgião acrescenta que a redução dos pequenos lábios é um procedimento de cunho estético e que visa melhorar a confiança e autoestima das pacientes. Além disso, afirma que, em mais de 95% dos casos, seria usada “para consertar assimetrias entre os lábios da vagina e não está ligada a alguma anormalidade, mas a questões estéticas” e ao desejo de “deixar a vagina com aspecto bem simétrico, com os pequenos lábios bem fechados” (SBCP, 5 nov. 2012, s.p.).

A raridade das referências no *site* da SBCP e até o fato de que apenas matérias da imprensa sobre o assunto são apresentadas, indicam que não tem havido interesse por parte dessa sociedade em se posicionar oficial e publicamente sobre o assunto. É possível estabelecer certo contraste com o material encontrado no *site* da Febrasgo, que apresenta indicativos incisivos da posição dessa instituição. Sobretudo o posicionamento adotado nos trabalhos publicados, a inserção institucional das/os autoras/es e outras escolhas estratégicas, como veremos, apontam para outra direção, se comparada com aquela que parece ser a adotada pela SBCP.

De acordo com as informações em sua página oficial, a Febrasgo^4^ é a principal referência no campo da ginecologia no Brasil, congrega os profissionais da área e também produz diretrizes a seguir. A busca pelos termos cirurgia íntima, cirurgia dos pequenos lábios, ninfoplastia e labioplastia resultou em três ocorrências. Trata-se de artigos, escritos por ginecologistas integrantes da associação, que refletem sobre o tema por meio das referências científicas do campo, indicando devidamente suas fontes e fundamentações.

O primeiro data de 21 de junho de 2017, é escrito por Lucia Alves da Silva Lara (21 jun. 2017)^5^ e se intitula “Cirurgia íntima: indicação e resultados”. O artigo começa definindo as cirurgias íntimas ou plásticas genitais como “procedimentos cirúrgicos na genitália com o objetivo de melhorar a estética”, especialmente no que se refere às preocupações das mulheres com “assimetria e hipertrofia dos pequenos lábios, flacidez de pequenos e grandes lábios, defeitos no introito vaginal, excesso de tecido adiposo suprapúbico, e dificuldade para obter satisfação sexual relacionada com a aparência da genitália”. Logo de início, anuncia que “a indicação médica para a cirurgia genital esbarra em questões legais e éticas já que não existem diretrizes que suportem a realização deste procedimento para modificação da anatomia genital. Isto indica que o procedimento cirúrgico só poderá ser realizado para os casos de alteração da função genital” (Lara, 21 jun. 2017, s.p.).

Considerando, contudo, que “a demanda da mulher existe” é preciso que sejam observadas as condições que levariam à indicação da cirurgia, conforme o que foi preconizado pelo American College of Obstetricians and Gynecologists (Acog, 2007). Essa instituição de referência na área defende que o procedimento só poderia ser realizado por ginecologistas e apenas em mulheres maiores de 18 anos que “manifestam desconforto psíquico e funcional com sua genitália” (s.p.). Conforme Lara (21 jun. 2017, s.p.), a indicação só aconteceria nos seguintes casos:

1) hipertrofia ou assimetria dos pequenos lábios percebida pela mulher que causa desconforto com atividades esportivas ou uso de roupas, dor ou aprisionamento intravaginal dos pequenos lábios durante a penetração vaginal; 2) alterações genitais devidas à gravidez ou à lesão obstétrica que afetam a aparência da genitália ou que interferem na sensação prazerosa ao coito; 3) frouxidão vaginal pós-parto que interfere na satisfação sexual da mulher.

Além disso, Lara (21 jun. 2017, s.p.) pondera que:

Até o momento, não existem diretrizes para nortear este procedimento quanto a técnica cirúrgica, dados de segurança, resultados anatômicos e funcionais. Também não existem critérios para definir a hipertrofia labial, e faltam estudos prospectivos e randomizados para definição da técnica cirúrgica ideal.

E acrescenta que os resultados cirúrgicos obtidos até o momento foram baseados em estudos retrospectivos e casos clínicos, sem a utilização de instrumentos validados, restringindo-se a apontar a “satisfação” com o resultado relatada pelas mulheres operadas. E, ao finalizar o trabalho, a ginecologista sintetiza, mais uma vez que:

Em resumo, faltam evidências quanto à indicação da cirurgia, sobre as técnicas mais indicadas, sobre resultados satisfatórios, e complicações cirúrgicas. Isto deriva em parte da ausência de critérios para definir se os aspectos anatômicos da vulva são normais ou não. ... É necessário discutir com a mulher as vantagens e desvantagens de cada técnica e possíveis resultados estéticos e complicações em curto e longo prazo. Assim, a indicação da cirurgia é pautada na queixa da mulher que pode ser realizada seguindo, rigorosamente, os princípios da ‘boa prática’, pois nem sempre o que a mulher pretende pode ser feito, e nem sempre as expectativas dela serão atingidas (Lara, 21 jun. 2017, s.p.).

Trata-se, portanto, de uma postura cautelosa e crítica em relação à natureza da demanda por parte das mulheres, ao avanço no aumento desse tipo de cirurgia e à precariedade dos estudos nesse campo.

Essa mesma autora, em colaboração com Neila Speck,^6^ no ano seguinte, publica o artigo “Cosmiatria genital” (Speck, Lara, 24 abr. 2018), no qual as autoras alertam para “o crescimento na oferta de cirurgias estéticas ginecológicas com a promessa de promover melhora da aparência e tornar a vida sexual da mulher mais prazerosa”. É interessante que, nesse caso, o foco recai ainda mais sobre a oferta de variados tipos de procedimentos. Segundo as autoras, multiplicam-se não somente as técnicas de intervenção possíveis (botox, preenchimento, clareamento, lipoaspiração, ninfoplastias, *piercing*, tatuagens), bem como os próprios termos utilizados, para os quais chamam a atenção: “Embelezamento íntimo, estética genital, estética vaginal, cirurgia íntima, bioplastia íntima, cirurgia estética genital, medicina estética e ginecologia, ginecologia estética, estética íntima, plástica da intimidade, cirurgia da intimidade, cirurgia cosmética vaginal etc.” (Speck, Lara, 24 abr. 2018, s.p.).

Alertam para o fato de que alguns procedimentos são oferecidos “sem qualquer evidência científica de resultados positivos na saúde sexual, o que infringe [a] ética médica”. Outros têm comprovação, mas são usados “com propagandas enganosas, e não aplicados por médico ginecologista”. A demanda por parte das mulheres não pode servir de justificativa para realizar procedimentos e *marketing* de práticas ainda pouco estudadas, a respeito das quais não se conhecem “taxas de complicações, parâmetros de sucesso do procedimento, ou definições de um resultado cirúrgico bem-sucedido” (Speck, Lara, 24 abr. 2018, s.p.).

Essa posição mais crítica também pode ser vista na avaliação que fazem em relação aos modelos de “embelezamento” que estão sendo propostos, o que qualificam como “farsa”:

As propagandas para atrair as mulheres, os padrões oferecidos prometem uma genitália com aspecto muito jovem, quase infantil, além da promessa da perfeição sexual [a] que estes procedimentos possam levar. Padrão de beleza da genitália não está estabelecido e é muito variável, dependente de uma série de fatores, culturas, religião, sexo, moda, assim como a resposta sexual feminina, que depende de fatores biológicos, psíquicos, culturais, relacionais, hormonais etc. Desta forma, prometer a ‘perfeição’ para beleza genital, e garantir que isto vai melhorar a vida sexual é uma farsa (Speck, Lara, 24 abr. 2018, s.p.).

E, por fim, é importante notar que anunciam um posicionamento como representantes da própria Febrasgo, em relação ao papel imprescindível do/a ginecologista no que se refere a essas intervenções:

Nós, Febrasgo, como representantes da ginecologia e obstetrícia do nosso país, queremos estabelecer diretrizes para que estes atos devam ser aplicados por médicos especialistas da nossa área. Estas mulheres necessitam de uma avaliação ginecológica apurada, com rastreio de neoplasias, estabelecimento de condições hormonais, avaliação de queixas sexuais, exclusão de infecções secundárias, avaliação do estado urodinâmico, para ver a indicação real do método, condições estas que só podem ser estabelecidas por médico ginecologista (Speck, Lara, 24 abr. 2018, s.p.).

O terceiro artigo publicado no *site* da Febrasgo, de autoria de José Humberto Belmino Chaves (23 fev. 2018),^7^ também chama a atenção acerca das controvérsias envolvidas nas cirurgias íntimas, como se pode ver pelo título: “Embelezamento no trato genital inferior feminino: qual o limite ético?”. O autor parte da constatação do crescente aumento da oferta de cirurgias estéticas vulvares e/ou vaginais, cujas primeiras referências datam dos finais da década de 1970, e lamenta que não seja acompanhado por um referencial teórico que sustente tal prática. Também indica a profusão de termos (“estética genital feminina”, “estética vaginal”, “cirurgia íntima”, “bioplastia íntima”, “cirurgia estética genital”, “medicina estética e ginecologia”, “ginecologia estética”, “estética íntima”, “plástica da intimidade”, “cirurgia da intimidade”, “cirurgia cosmética vaginal” etc.) e de procedimentos encontrados na internet (“técnicas”, “tratamentos” [estéticos ginecológicos], “correção”, “remodelagem”), segundo o médico “encarados como ‘alternativa’, além de identificados como simples e ‘rápidos’”. Esta “profusão de designações”, segundo ele, seria sintomática de que “algo está errado neste cenário”, o que suscitaria certa “inquietação ética”. E, além disso: “Fazem-se afirmações sem qualquer suporte científico, garantem-se resultados improváveis. Mais, cria-se a necessidade, inventa-se a ‘doença’ ou defeito e oferecem a sua cura mágica. Exploram-se as fraquezas individuais: cirurgias que melhoram a autoestima, a função sexual, que salvam casamentos” (Chaves, 23 fev. 2018, s.p.).

Dos procedimentos, o autor cita: o aumento dos lábios vaginais, a diminuição/redução dos pequenos lábios vaginais (chamada ninfoplastia ou labioplastia), correção da dilatação da vagina, eliminação/enxerto da gordura do púbis, reconstrução do hímen (himeoplastia), remodelagem do clitóris (clitoriplastia), reconstrução do períneo (perineoplastia), ampliação do ponto G. E menciona que são utilizados instrumentos tecnológicos variados como *lasers* e injeções de colágeno/ácido hialurônico/polimetacrilmetacrilato ou mesmo a chamada “‘Terapia Drácula’ para o rejuvenescimento da vagina” (destaque no original), que utiliza injeções de plasma rico em plaquetas. E logo a seguir acrescenta que “alguns autores sugerem que este campo gravite perigosamente próximo da mutilação genital feminina” (Chaves, 23 fev. 2018, s.p.).

Chaves (23 fev. 2018) acrescenta que a literatura menciona a procura de cirurgiões plásticos por parte das mulheres para realizar esses procedimentos com fins estéticos, sem apresentar, contudo, os riscos, complicações ou sequelas e mesmo sem nunca fazer a “ressalva que pode não acontecer a magia prometida”. Relata que a labioplastia tem sido uma das intervenções mais procuradas no Brasil e sugere que a justificativa para tal busca pelas correções de possíveis “imperfeições” tenha a ver com a interferência da indústria pornográfica e sua exigência e projeção de um “padrão estético uniforme”, que se expressa na escolha por “uma vulva sem nenhum pelo, com coloração rosada, grandes lábios gordinhos e firmes, pequenos lábios discretos e clitóris sem projeção. Numa perspectiva de adquirir um formato do genital com semelhanças idênticas à fase que se apresenta na menina pré-púbere” (s.p.).

O autor também refere uma preocupação especial com as adolescentes, que têm sido retratadas na literatura como faixa etária mais vulnerável “a optar por esse mantra ‘embelezamento íntimo’” (destaque no original). Por meio da “comparação com as amigas”, da “tirania da beleza adotada em filmes pornográficos”, ou mesmo pela “interferência de familiares”, em um período de “imaturidade psicológica”, elas buscam uma transformação que poderá ter consequências futuras. Chaves (23 fev. 2018, s.p.) aponta uma “indefinição fisiológica das estruturas dos genitais pela ação da puberdade” e que modificações indevidas podem ocasionar “perdas da arquitetura que repercutirão com gravidezes e os partos e de forma progressiva com a chegada da menopausa”.

Especificamente no que diz respeito ao argumento usado para justificar as intervenções de que visariam à “saúde biopsiquisocial”, Chaves (23 fev. 2018, s.p.) pondera que a discussão central deve ser acerca da “busca em ter uma aparência genital semelhante a um modelo pré-concebido, como solução para corrigir uma frustração”, o que pode não ser resolvido com a cirurgia. Lembra também as consequências imprevistas com a cirurgia na vulva, na medida em que pode haver interferência na inervação e na capacidade sensitiva e erétil dos tecidos.

Em razão desse tipo de preocupações, o autor sustenta que

[n]enhum procedimento no trato genital inferior feminino deve ser efetuado sem que previamente um médico habilitado, preferencialmente com título de especialista em Ginecologia-Obstetrícia, faça uma avaliação para interpretar globalmente a clínica da paciente. Assegurando-se de que a queixa apresentada efetivamente necessite de uma intervenção cirúrgica, ou se trata por exemplo de um problema com outras dimensões da esfera psicológica (Chaves, 23 fev. 2018, s.p.).

Refere-se ainda às recomendações da Society of Obstetricians and Gynaecologists of Canada publicadas em 2013 (SOGC, 2013), para reafirmar o papel do ginecologista. Caberia a esse especialista não apenas realizar o exame e histórico clínico da paciente do ponto de vista ginecológico e sexual, “procurando excluir disfunções sexuais ou psicológicas e eventual coerção”, mas também educar as mulheres no que se refere à sua anatomia e variantes fisiológicas da genitália. Além disso, deveria alertar para a falta de dados e evidências científicas relativas a essas práticas, que não poderiam ser realizadas antes de atingida a maturidade sexual. Chaves (23 fev. 2018), por fim, apela para a importância de se discutir a relação entre ética e estética, advogando a importância de que o tema seja debatido nas instituições médicas, para que se possa tomar uma posição “consciente e informada” (s.p.).

Ainda considerando o âmbito da Febrasgo, no início de 2019 se inicia a publicação da revista *Ela*, tanto no *site* quanto impressa, com tiragem de 15 mil exemplares, tendo como jornalista responsável Cássia Fragata. Segundo a proposta editorial, expressa nas palavras do então presidente da instituição, César Eduardo Fernandes, trata-se de um canal de comunicação que pretende “conversar diretamente” com as mulheres de qualquer idade, focalizando “temas do universo feminino”, como “prevenção, tratamentos, sexualidade, nutrição, bem-estar”, além de lazer e cultura, “sempre com a qualidade e a credibilidade da Febrasgo” (Fragata, 2019, p.3). Esse número é especialmente interessante pelo fato de que a matéria em destaque na capa é “Estética íntima: os perigos e os cuidados com cirurgias e procedimentos cosméticos vulvovaginais”. Ou seja, a Febrasgo selecionou este tema como relevante para a chamada do número inaugural de sua nova revista.

No corpo da revista, a reportagem, que assim como a capa é ilustrada por diferentes imagens de orquídeas (padrão comum para aludir à genitália feminina), é encabeçada pelo título “Viva a Diferença! Muitos são os modismos que surgem no universo feminino. Alguns vêm pra ficar e outros passam como chuva de verão”. A introdução chama a atenção para a saúde e para a necessidade de se tomar cuidado em relação “à nova moda das cirurgias e procedimentos cosméticos vulvovaginais” (Fragata, 2019, p.23). Cita as técnicas como preenchimento, *peeling*, diminuição e aumento dos grandes lábios, que podem ter consequências sérias para as mulheres. Reforça que não se deve acreditar em “tratamentos milagrosos” e questiona “sobre a verdadeira necessidade de passar por algum desses procedimentos”, enfatizando que o ginecologista deve ser o primeiro profissional a ser consultado (p.23). No subitem “Estética X saúde”, a ginecologista Neila Speck fala sobre as razões da procura por esse tipo de procedimentos, de forma enfática: “É o sonho da vagina da Barbie, e isso não existe, é um modismo sem fundamento científico” (p.23):

Algumas mulheres podem precisar de intervenção, mas aí não se trata de estética, mas sim de uma cirurgia funcional que vai melhorar a vida dessa pessoa. ... Se não há um padrão de beleza definido para o rosto, existe um padrão de beleza definido para a região genital? Não. As pessoas estão fantasiando, à procura de subterfúgios para encontrar a felicidade por meio de uma mudança estética (Fragata, 2019, p.24).

Considera-se, assim, a existência de algumas “alterações anatômicas” mais significativas que podem causar incômodos e que justificariam uma intervenção como a ninfoplastia, por exemplo. Mas quando se trata de uma cirurgia por motivos estéticos, é necessário levar em conta as possíveis implicações devidas à intervenção em uma região delicada e também ter cuidados em relação às promessas feitas de deixar a região “mais bonita” e de melhora na performance sexual. Mais uma vez, conforme o depoimento de Speck, citada na matéria,

isso é enganoso e é uma prática altamente condenável ... Esse tipo de procedimento estético não vai interferir na libido nem nos problemas sexuais dessa mulher. Ela vai trocar uma região vaginal boa, natural, por uma ruim, com uma cicatriz (Fragata, 2019, p.25).

O último tópico aborda “a beleza da diversidade” em geral e o fato de que as mulheres não precisariam “ser iguais para ser bonitas”. O mais importante seria valorizar a autoestima, inclusive em relação aos parceiros, já que estes “vêm e vão e, certamente, se o relacionamento não está bom, um procedimento estético não vai mudar o que se sente um pelo outro” (Fragata, 2019, p.25). Conforme a ginecologista entrevistada: “Já vi casais discutindo isso, mas na verdade percebe-se claramente que o relacionamento não está sólido. E, se a mulher tem a autoestima lá embaixo, acaba concordando em fazer alguma coisa, acreditando que o parceiro vai voltar a ser legal”. Por fim, a reportagem acaba com uma mensagem de incentivo: “Então, menina, curta-se como é! Você é forte e poderosa! Vá em frente, com graça e muito charme!” Embora haja uma tentativa de valorização da diversidade e da autoestima feminina, não se abre mão, pelo menos, da “graça e muito charme”, adjetivos tradicionalmente associados ao universo feminino (p.25).

Esse tom crítico aos procedimentos estéticos na genitália feminina e a insistência na importância do papel do/a ginecologista assumido pela Febrasgo, a julgar pelos documentos apresentados, revelam uma tentativa de delimitação das fronteiras nesse campo de atuação. É bom lembrar que essa abordagem mais crítica a novos procedimentos e tratamentos não necessariamente é uma posição generalizada no âmbito dessa instituição, como pode ser percebido, por exemplo, na sua atuação quanto às controvérsias relativas aos contraceptivos hormonais e seus impactos na vida e saúde das mulheres (Klöppel, 2021). No cenário das cirurgias estéticas íntimas e das contraposições aos/às cirurgiões/ãs plásticos/as, pelo menos de parte dos/as especialistas que têm se manifestado e representado a instituição nesse assunto, parece, contudo, haver uma preocupação com os “excessos” que vêm ocorrendo e, muitas vezes, com a falta de participação de ginecologistas nesse cenário.

## A produção de novas referências

Um último conjunto de material empírico talvez ajude a visualizar o fato de que, a despeito da posição dos ginecologistas citados, o campo vem sendo majoritariamente dominado pelo discurso e pela prática dos/as cirurgiões/ãs plásticos. Se considerarmos a publicação de livros de referência, a abordagem predominante defende a realização das cirurgias estéticas íntimas.

Só muito recentemente são publicados compêndios mais abrangentes sobre o tema da cirurgia genital feminina, tanto no exterior como no Brasil. Os quatro principais livros identificados expressam já nas suas páginas iniciais o reconhecimento de que esse é um campo de crescimento enorme e muito recente. E também que haveria pouca bibliografia de referência sobre o assunto, o que justificaria a publicação das obras. Outra característica comum é a menção ao fato de que as mulheres estariam cada vez mais interessadas nesse tipo de cirurgia, e caberia à medicina oferecer respostas e recursos atualizados.

O primeiro livro, editado em 2016, é organizado por Michael P. Goodman, ginecologista especializado em cirurgia estética genital, e mais 11 colegas, e se intitula *Female genital plastic and cosmetic surgery* (Goodman et al., 2016). A apresentação do volume menciona que a cirurgia plástica genital feminina tornou-se muito procurada por mulheres que buscam “melhora” na aparência genital, alívio do desconforto e aumento do prazer sexual. Essa demanda pode ser abordada por meio de cirurgias que combinem procedimentos ginecológicos, plásticos e cosméticos. E, para tanto, há uma demanda crescente de médicos adequadamente treinados e capazes de realizá-las. Os 21 capítulos escritos por especialistas reconhecidos na área abrangem temas como: anatomia; procedimentos cirúrgicos específicos e todas as suas variações; justificativas das pacientes para a cirurgia; diretrizes de treinamento e questões éticas; estatísticas de resultados; questões sexuais; seleção de pacientes; riscos e complicações potenciais. Salienta-se que contém a descrição das técnicas cirúrgicas passo a passo e desenhos e fotografias que são apresentados como compreensíveis e inequívocos.

Em 2017 é publicado *Female cosmetic genital surgery: concepts, classification and techniques*, de Christine A. Hamori (cirurgiã plástica), Paul E. Banwell (cirurgião plástico) e Red Alinsod (ginecologista especializado em cirurgia), obra traduzida e publicada no Brasil em 2018 (Hamori, Banwell, Alinsod, 2017). O livro é dividido em três partes: introdução, técnicas e avanços e conta com prefácio de D.J. Hodgkinson, autor do artigo inaugural escrito em 1984 sobre o tema. No prefácio, Hodgkinson conta que, em 1984, não poderiam imaginar que o procedimento viria a ser tão aceito, como nos dias atuais, e que passaria a ser concebido como um procedimento estético. Nos capítulos que compõem a parte introdutória, destaca-se a influência das imagens de genitálias na internet e em outros meios, bem como de novas técnicas de depilação que levariam as mulheres à busca do aprimoramento estético de suas vulvas. Informa-se, mais uma vez, que o padrão de normalidade é muito variável, mas isso não impede que as mulheres procurem melhorias por meio das intervenções cirúrgicas. Caberia aos médicos informar sobre os procedimentos, discutindo riscos e objetivos apresentados como realistas que iriam na direção do que chamam de harmonização da genitália mais do que a busca por medidas precisas. Há ainda a menção à questão da mutilação genital feminina e a procura de estabelecer fronteiras bem definidas entre ela e as cirurgias estéticas realizadas em lugares como EUA, Inglaterra e Austrália, onde as mulheres teriam a livre escolha assegurada.

No livro *Aesthetic vaginal plastic surgery: a practical guide*, a cirurgiã plástica colombiana Lina Triana (2019) também defende a importância dos tratamentos cirúrgicos estéticos para a região genital feminina. Chama a atenção, inclusive, o fato de a autora usar a categoria “rejuvenescimento” vaginal e genital para se referir ao conjunto de procedimentos que propõe. Assim, como outros/as autores/as, reporta sua história de reconhecimento nesse campo, desde meados dos anos 2000, quando identificou a necessidade de ajudar suas pacientes que demandavam melhoria de sua estética íntima. Triana é enfática ao argumentar que a vagina representa o poder e o mistério femininos e por isso é tão valorizada em nossa sociedade. E que graças à quebra de certos tabus é possível realizar intervenções também nessa parte do corpo feminino. Além disso, traz um comentário que passa a ser comum entre os/as autores/as, que é a comparação com a cirurgia de próteses nos seios. Afirma que nos anos 1970 esse era um procedimento polêmico, cuja aceitação era questionável, o que já não aconteceria mais. E preconiza que o mesmo deve acontecer com a cirurgia genital feminina, mais um procedimento a ajudar as mulheres a conseguir seu bem-estar.

Em 2018 há também a publicação do volume *Cirurgia íntima: plástica genital feminina*, organizado pelo cirurgião plástico brasileiro André Colaneri (2018), que inclui textos de outros/as 12 autores/as. Segundo o organizador, o livro seria o “primeiro a ser escrito em língua portuguesa sobre o tema” e viria a “preencher a lacuna da literatura especializada sobre o assunto” (s.p.). O autor também cita o grande aumento nas cirurgias estéticas íntimas, motivado pela procura por parte das mulheres, o que leva à necessidade de aprofundamento no tema. Conta que a cirurgia plástica no geral e agora a cirurgia estética genital sofreram preconceito, inclusive por parte de colegas médicos, mas argumenta que a cirurgia estética pode proporcionar bem-estar emocional, autoestima e qualidade de vida às pacientes. Dessa forma, a cirurgia estética vem sendo valorizada por poder “modificar e adequar desarmonias corporais” (s.p.).

O percurso argumentativo do autor ilustra a abordagem que vai se tornando cada vez mais acentuada no campo. Nela, a dimensão estética, agora traduzida pela ideia de “harmonia”, já que não há consenso sobre classificações e medidas da anatomia genital feminina (Rohden, Cavalheiro, 2021), associada à demanda por “bem-estar” e “satisfação”, sustenta as justificativas dos/as médicos/as para a intervenção cirúrgica. Com base nessas redefinições, assistimos a um enfraquecimento da dicotomia inicialmente presente no campo, entre intervenções de cunho estético e funcional e um consequente alargamento das possibilidades de intervenção.

## Considerações finais

O conjunto das informações apresentadas até aqui mostra um cenário de disputas entre ginecologia e cirurgia plástica na controversa cirurgia estética genital. Certamente, disputas e controvérsias entre especialidades médicas em torno do corpo feminino não são uma novidade (Martin, 1992; Groneman, 1994; Russett, 1995; Moscucci, 1996; Wijngaard, 1997; Laqueur, 2001; Rohden, 2001). No terreno das cirurgias íntimas, contudo, é possível identificar novos elementos que passam a compor um quadro bastante complexo.

Um dos tópicos centrais diz respeito ao debate acerca de se a questão seria de ordem funcional, privilegiada por ginecologistas, ou estética, do âmbito dos/as cirurgiões/ãs plásticos/as – tensão que já se apresentava nos trabalhos de referência pioneiros no campo e que se mantém quase que exclusivamente no discurso dos/as ginecologistas. Já entre os/as cirurgiões/ãs plásticos/as, essa dicotomia vai se dissolvendo por meio de uma ênfase na ideia fluida e pouco aprofundada de que incômodos com a aparência da genitália podem trazer insatisfação e problemas na autoestima que justificariam uma intervenção. Do ponto de vista desses/as especialistas, as cirurgias seriam seguras, rápidas e eficazes, trazendo resultados satisfatórios, e os procedimentos vão sendo, então, cada vez mais naturalizados (Rohden, 2021).

Para discutir o processo de naturalização desse fenômeno, três argumentos podem ser acionados. O primeiro deles diz respeito à hipótese de que esse clima propício a intervenções, tais como a cirurgia íntima, só se torna possível em um contexto no qual a lógica do aprimoramento de si se transforma em um imperativo, incentivado pelo acesso a diferentes tipos de tecnologias e recursos biomédicos. A busca insistente por recursos que levem à otimização e ao aprimoramento expressaria, inclusive, novos modos de subjetivação, característicos da contemporaneidade (Rose, 2007; Clarke et al., 2010; Dumit, 2012). E como dizem os/as cirurgiões/ãs plásticos/as, nada mais natural do que as mulheres procurarem aprimorar até mesmo o desenho das suas genitálias. Cabe ainda mencionar o efeito que as redes sociais vêm tendo na conformação de novos imperativos de transformação corporal. O compartilhamento de imagens, incluídas as ilustrativas do “antes e depois” de uma cirurgia, tem se tornado comum e impactado as expectativas em relação à possibilidade de investimentos em um corpo ideal. A procura pelas cirurgias íntimas precisa também ser lida à luz desse processo.

Ao lado disso, temos a própria conformação de um novo enquadramento para as intervenções biomédicas desenvolvido em um longo processo que culmina no que Clarke e colegas (2010) chamam de biomedicalização da sociedade. Em particular, faz sentido aqui recuperar o que dizem acerca de mais plasticidade dos corpos, que passam a se tornar matéria suscetível a muitas formas de intervenção e manipulação, na direção de customização e aprimoramento potencialmente incessantes. Nesse plano, algumas intervenções biomédicas deixam de estar associadas a problemas de saúde e passam a incorporar prioritariamente a busca por melhoria e alta *performance* (Conrad, 2007). A saúde pode não ser a prioridade diante da lógica do aprimoramento (Edmonds, Sanabria, 2014, 2016; Rohden, 2017).

As cirurgias íntimas, e as justificativas acionadas pelos/as seus/suas defensores/as, parecem ser uma ilustração muito contundente dessa busca por aprimoramento que atingiria todas as partes do corpo e também um retrato dessa percepção de uma plasticidade corporal. O que está em cena é a compreensão de que os corpos estariam disponíveis a todo tipo de transformação e de que a anatomia sempre teria algo a ser modificado ou melhorado. Não estaríamos nos limites do uso de recursos biomédicos visando à resolução de problemas de saúde, mas no cenário de novas exigências encarnadas nos desenhos corporais “aperfeiçoados”.

Por fim, não se pode deixar de mencionar a centralidade do dispositivo de gênero no contexto das cirurgias genitais femininas. O que os discursos favoráveis ao procedimento atestam é uma delimitação extremamente marcada das fronteiras entre masculino e feminino, traduzida em diferenças idealizadas em corpos binariamente distintos. Além disso, reforçam a insistência em constante autovigilância por parte das mulheres que deveriam estar atentas aos padrões considerados mais adequados ou “femininos” e efetivar as transformações necessárias para se aproximar desses modelos. Aquilo a que assistimos, então, no caso dessas práticas, pode ser lido como mais um exemplo da vigorosa recitação do gênero nos corpos, em sua matriz binária e heteronormativa, como diria Butler (1993). Um exemplo que traduz um longo processo histórico de normalização de muitas diferenças sociais que acabam por ser inscritas diretamente nos corpos (Nurka, 2019).

Por meio dessas pequenas incisões na carne, quase que literalmente, vão se construindo novos corpos e novos padrões do que seria uma natureza redefinida e redesenhada, a representar um feminino idealizado. E, a julgar pelas posições proeminentes expressas no campo e aqui ilustradas, nada dessa complexidade tem afetado a prática dos/as cirurgiões/ãs que têm garantido a expansão desse campo de intervenções.

## Data Availability

Não estão em repositório.

## NOTAS

1 Talita Franco formou-se na Faculdade Nacional de Medicina da Universidade do Brasil, em 1964, e foi professora titular de cirurgia plástica do Hospital Universitário Clementino Fraga Filho, da Faculdade de Medicina da Universidade Federal do Rio de Janeiro, e membro titular da Academia Nacional de Medicina. Oriunda de uma família de médicos (desde o século XIX) e mãe de Diogo Franco, também cirurgião plástico, coautor do artigo.

2 Nessa e nas demais citações de textos publicados em outros idiomas, a tradução é livre.

3 Conforme apresentado no *site* da SBCP, disponível em: http://www2.cirurgiaplastica.org.br/sbcp/missao/. Acesso em: 28 maio 2020.

4 Conforme informações disponíveis em: https://www.febrasgo.org.br/pt/institucional/a-febrasgo. Acesso em: 28 maio 2020.

5 Lucia Alves da Silva Lara é médica ginecologista e obstetra especialista em sexualidade humana, mestre e doutora pela Universidade de São Paulo (USP) em tocoginecologia, presidente da Comissão Nacional de Sexologia da Febrasgo, coordenadora do Ambulatório de Estudos em Sexualidade Humana da Faculdade de Medicina de Ribeirão Preto, da USP.

6 Ginecologista e obstetra, com qualificação em patologia do trato genital inferior e colposcopia, habilitação em cirurgia a laser, presidente da Comissão Nacional Especializada do Trato Genital Inferior da Febrasgo.

7 Professor titular de ginecologia da Universidade Estadual de Ciências da Saúde de Alagoas e professor adjunto de ginecologia da Faculdade de Medicina da Universidade Federal de Alagoas, além de vice-presidente da Associação de Patologia do Trato Genital Inferior e Colposcopia.
